# New tools for studying osteoarthritis genetics in zebrafish

**DOI:** 10.1016/j.joca.2012.11.004

**Published:** 2013-02

**Authors:** R.E. Mitchell, L.F.A. Huitema, R.E.H. Skinner, L.H. Brunt, C. Severn, S. Schulte-Merker, C.L. Hammond

**Affiliations:** † Department of Biochemistry, University of Bristol, Bristol BS8 1TD, UK; ‡ Department of Physiology & Pharmacology, University of Bristol, Bristol BS8 1TD, UK; § Hubrecht Institute, KNAW & UMC, Utrecht 3584 CT, The Netherlands; ‖ EZO, Wageningen University, The Netherlands

**Keywords:** Zebrafish, Gene expression, Cartilage, Bone, Model, Osteoarthritis

## Abstract

**Objective:**

Increasing evidence points to a strong genetic component to osteoarthritis (OA) and that certain changes that occur in osteoarthritic cartilage recapitulate the developmental process of endochondral ossification. As zebrafish are a well validated model for genetic studies and developmental biology, our objective was to establish the spatiotemporal expression pattern of a number of OA susceptibility genes in the larval zebrafish providing a platform for functional studies into the role of these genes in OA.

**Design:**

We identified the zebrafish homologues for *Mcf2l, Gdf5, PthrP/Pthlh, Col9a2,* and *Col10a1* from the Ensembl genome browser. Labelled probes were generated for these genes and *in situ* hybridisations were performed on wild type zebrafish larvae. In addition, we generated transgenic reporter lines by modification of bacterial artificial chromosomes (BACs) containing full length promoters for *col2a1* and *col10a1.*

**Results:**

For the first time, we show the spatiotemporal expression pattern of *Mcf2l*. Furthermore, we show that all six putative OA genes are dynamically expressed during zebrafish larval development, and that all are expressed in the developing skeletal system. Furthermore, we demonstrate that the transgenic reporters we have generated for *col2a1* and *col10a1* can be used to visualise chondrocyte hypertrophy *in vivo*.

**Conclusion:**

In this study we describe the expression pattern of six OA susceptibility genes in zebrafish larvae and the generation of two new transgenic lines marking chondrocytes at different stages of maturation. Moreover, the tools used demonstrate the utility of the zebrafish model for functional studies on genes identified as playing a role in OA.

## Introduction

Osteoarthritis (OA) is an increasingly common degenerative joint condition, estimated to affect more than 100 million people worldwide and more than 40% of people over 70 years of age^20^. OA has a complex, multifactorial aetiology and treatment options remain limited; however there is increasing evidence of a genetic component to OA (reviewed by Cornelis et al^2^). It has been estimated from a number of twin studies that the genetic contribution to OA is between 39% and 60% in hip and knee OA, respectively^3,4^. To date, however, while many genes have been shown to be differentially expressed between osteoarthritic and healthy chondrocytes by RT-PCR and microarray analyses^5,6,7^ relatively few genes have been identified through association studies to have reached genome wide significance; those that have include Growth/Differentiation factor (GDF5), a cluster of six genes on 7q22 (comprising PRKAR2B, HPB1, COG5, GPR22 DUS4L and BCAP29) and recently MCF2L^8,9,10^.

There is increasing evidence showing that there are significant similarities between OA progression and the normal developmental process of endochondral ossification, whereby a cartilage template is progressively replaced by bone (reviewed by Pitsillides and Beier^11^). Endochondral ossification occurs during embryonic development and continues postnatally in the cartilage growth plates of long bones, which give the potential for continued skeletal growth. In the cartilage growth plate, chondrocytes are organised into zones of progressive maturation beginning with the metabolically inactive resting chondrocytes, which become activated and proliferate; following proliferation they enter a pre-hypertrophic and finally a hypertrophic state (reviewed by Mackie et al^12^). Hypertrophic chondrocytes, which are regarded as terminally differentiated, remodel their matrix through expression of proteases such as Matrix metalloprotein 13 (MMP13) and secretion of different collagens, in particular Type X Collagen^13^.

Ectopic chondrocyte hypertrophy, marked by expression of Type X Collagen, is seen as a hallmark of OA in humans and in diverse animal models from mice to sheep (reviewed by van der Kraan and van den Berg^14^). Genetic changes that stimulate chondrocyte hypertrophy lead to increased incidence of OA^14^ raising the prospect that inhibition of chondrocyte entry to hypertrophy might be a therapeutic target in OA. Moreover, these parallels between endochondral ossification and OA raise the prospect that developmental models could be used to study the processes that lead to chondrocyte hypertrophy. Therefore, tools that allow investigators to follow the differentiation state of chondrocytes and the onset of hypertrophy in real time *in vivo* will be particularly valuable.

Zebrafish, along with another small teleost species medaka, have long been used as model organisms for developmental biology. They owe their popularity in part to the rapid external development of their larvae, to their amenability to genetic manipulation and also, importantly, to the translucency of the larvae, which allows detailed observation of organogenesis in the living fish. The zebrafish craniofacial skeleton is of comparable complexity to that of terrestrial vertebrates, and contains bones of both dermal and chondral origins, which form from neural crest-derived cells relatively early in development^15^. Importantly both the key regulators of skeletal development and the control of the major signalling pathways are highly conserved between mammals and teleosts^16^. As such, findings in fish are likely to be applicable to mammalian osteogenesis. Therefore, to increase the utility of the zebrafish model for functional *in vivo* studies into OA, we generated a new transgenic *col10a1* reporter line to enable monitoring of chondrocyte hypertrophy in live fish in real time.

We describe, for the first time in any animal model, the spatiotemporal expression of the OA associated gene *mcf2l* during early development. Additionally we describe zebrafish expression of parathyroid hormone–related protein (*pthrp)* and the mRNA expression of three key collagens and *gdf5* are shown. These tools provide a platform from which to probe the function of these genes during cartilage development *in vivo.*

## Materials and methods

### Zebrafish husbandry

Zebrafish were maintained as described^17^. All experiments on zebrafish were approved by the local ethics committee and the Home Office (Project licence number 30/2863).

### Skeletal staining

The protocol for bone and cartilage staining was as described previously^18^.

### *In situ* hybridization

As previously described^19^ *In situ* hybridisation probes previously used were *gdf5*^20^, *col10a1*^21^.

### Generation of new probes

Total RNA was extracted from zebrafish at 2 days post fertilization (dpf), 5 dpf and adult fin using EZNA total RNA kit following the manufacturer's protocol. 1 μg RNA was taken and used as a template to synthesize cDNA using promega MMLV-RT following the manufacturer's protocol. The cDNAs were pooled and 1 μg of the pooled cDNA was used as the template for a 50 μl PCR reaction using Roche Fidelity Taq (with conditions as per manufacturer's protocol) and a simple 35 cycle extension programme. Primers sequences used for probe synthesis are shown in Table I. The PCR product was cleaned with a PCR purification kit (E.Z.N.A) and 8 μl was used for RNA Dig labelled probe transcription as previously described^22^. To generate *mcf2l, col9a2*, and *col1a2* probes we amplified T3 polymerase tailed fragments from total cDNA and used the cleaned PCR product as a template for probe synthesis. The *pthrp* PCR product was cloned into pGEMT, digested with NdeI, and transcribed with primer T7 for antisense probe synthesis. Probes were synthesized as previous described^22^.

### Transgenic lines

The Tg(*Col2a1a*BAC:mcherry) stable line was generated by injection of the modified BAC construct previously described^18,23^ along with tol2 mRNA. Injected larvae were grown to sexual maturity and the F2 generation was screened for evidence of germline integration. The *osterix* reporter line Tg(OlSp7:mCherry)zf131 has been previously described^24^.

### Col10a1 transgenic line generation

The Tg(*Col10a1*BAC:mCitrine) transgenic line was generated following previously published protocols^25^. The BAC modified was DKEYP-115C4, the homology arm primers HA1 and HA2 were:

Forward primer: 5′-CTACATCATCACTTATAACTGTTGGAATTCTGTTTCAGATTTGACCTCAGACCATGGTGAGCAAGGGCGAGGAG-3’ and

Reverse primer: 5′-GCAGCCGTCAAGGCCACCAGGAGAAGAAGAATGCTTACTACTCGTAGTTCTCAGAAGAACTCGTCAAGAAGGCG-‘3.

The primers for amplification of the tol2 sites and for confirmation of integration have been previously published^25^. The stable *col10a1:citrine* reporter line was generated by injection of the modified BAC construct containing citrine under the control of the col10a1 promoter. Larvae showing strong mosaic transgene expression were grown to maturity and their offspring screened to identify germline carriers.

### Antibody labelling

Larvae were incubated with the following primary antibodies: anti-DSred clontech 1/200, anti-col2 II-II6B3 (D.S.H.B.) 1/200, anti-GFP (Abcam) 1/200 and anti-digoxigenin-rhodamine (Roche) 1/200) overnight at 4°C and then incubated with fluorescent conjugated secondaries (Dylight 488, and 550 1/500) for 3 h at RT, as previously described^18^. Larvae were viewed on a Leica SP5 confocal.

### Cryosectionning

For cryosectionning larvae were fixed, incubated in 20% sucrose overnight, snap frozen in OCT and 20 μM sections cut on a Leica cryostat at −20°C. Sections were rehydrated in PBS and viewed on a compound microscope.

### Phylogenetic analyses

Phylogenetic analyses were performed using the ‘Phylogenetic Tree’ web resource available from the Computation Biochemistry Research group at the Swiss Federal Institute of Technology (http://www.cbrg.ethz.ch/services/PhylogeneticTree)^26^. Sequences were input and analysis was run as in distance mode with the results displayed as an unrooted tree in the case of the mcf2l analysis and as a rooted tree for the pthlp analyses.

## Results

### Mcf2l expression

**Fig. 1:**
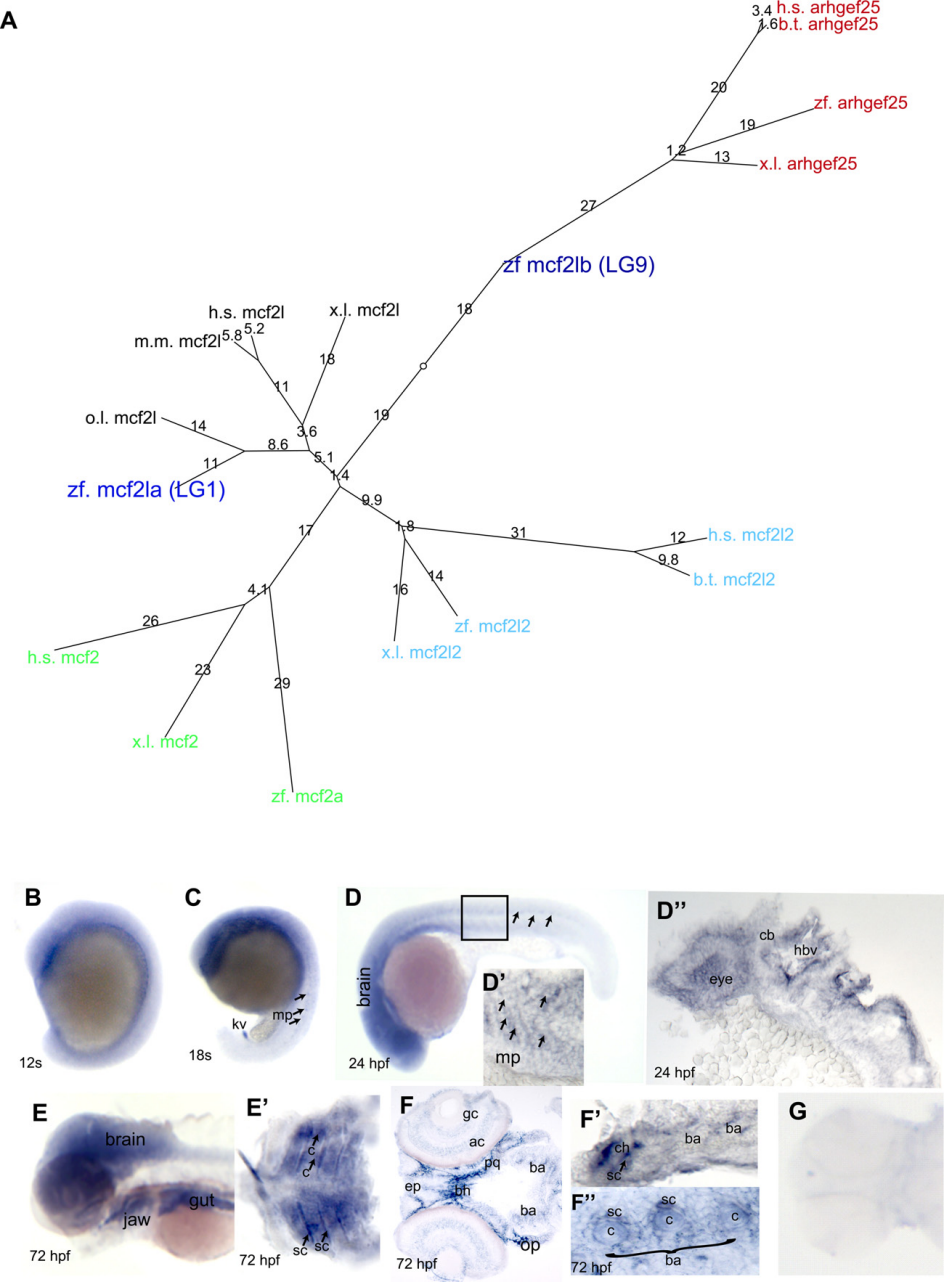
***Mcf2la is* dynamically expressed during zebrafish development** A phylogenetic tree to show that mcf2la clusters with mcf2l orthologues from vertebrate species, while mcf2lb is more derived. zf = zebrafish, ol = oryzias latipes {medaka}, bt = bos Taurus {cow}, hs = homo sapien {human}, mm = mus musculus {mouse}, xl = xenopus laevis {clawed frog}. B–F″ whole mount *in situ* hybridisation expression for *mcf2la*, B–E show whole mount images, while D′, D″, F–F″ show cryosections. 12G. Sense control for *mcf2la*. Anterior is to the left, with the exception of B–C where anterior is up. Ages are as follows 12s (B) 18s (C), 24 hpf (D–D″), 72 hpf (E–F″). Arrows in top panel indicate the muscle pioneers. Arrows in bottom panels point to chondrocytes (labelled c) or to cells surrounding the cartilage elements (sc).., ba = branchial arches kv = Kupffer'svesicle, jj = jaw joint, mp = muscle pioneers, cb = cerebellum, hbv = hindbrain vesicle, c = chondrocytes, op = operculum, pq = palatoquadrate, sc = surrounding cells (which include the perichondrial cells), ep = ethmoid plate, bh = basohyal, gc = ganglion cell layer, ac=amacrine cells.

The MCF2L locus has recently been identified as having a genome wide association with human hip/knee^8^. We identified two possible zebrafish homologues of Mcf2l (*mcf2l*) on LG1 and LG9 using BLAST searches. To establish which is the closest orthologue of mammalian Mcf2l we performed synteny analysis using Ensembl and generated a phylogenetic tree [Fig. 1(A)]; through these analyses we established that the *mcf2l* gene located on LG1 (which we denote *mcf2la*) is the closest orthologue of mammalian Mcf2l while the gene on LG9 likely arose through the ancestral genome duplication event in fish^27^ and is more distantly related to the mcf2l cluster. To establish the spatiotemporal expression of *mcf2la* we generated sense and antisense *in situ* probes. While the sense probe showed no expression [Fig. 1(G)], *mcf2la* was dynamically expressed during zebrafish development [Fig. 1(C–F″)]. At early stages *mcf2la* is expressed in the yolk syncytial layer [Fig. 1(B)]. At 18 somites (18 hpf), the strongest expression was seen in the Kupffer's vesicle [labelled kv in Fig. 1(C)]. Weaker expression was also seen throughout the brain, in the eye and in muscle pioneers from the 18 somite stage to 24 hpf [Fig. 1(D–D″)]. At 72 hpf, strong expression was observed in the ventral jaw elements and gut, along with expression in the brain and neural tube [Fig. 1(E)]. Dissection of the jaw elements revealed that *mcf2la* was expressed in cells surrounding the branchial arch cartilages, which include the perichondral cells, but may also include other cells found in close proximity [Fig. 1(E′–F″)]).

### *Pthlh/Pthrp* expression

**Fig. 2:**
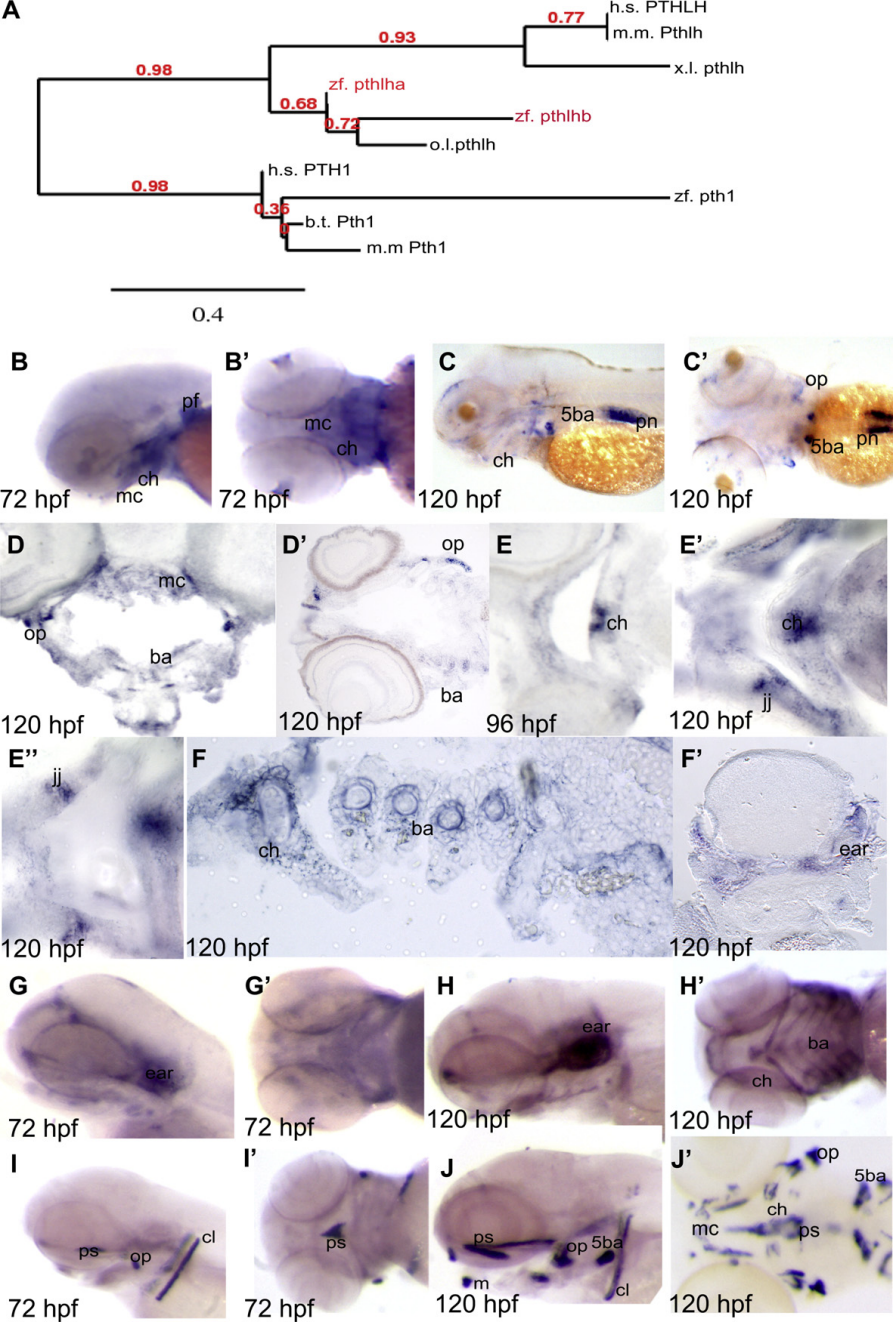
**mRNA expression of *pthlha, gdf5, col9a2* and *col10a1*** A phylogenetic tree to demonstrate that *pthlha* segregates with the vertebrate *Pthlh* genes h.s.=homo sapiens, m.m.=mus musculus, zf=zebrafish, x.l.=xenopus laevis, o.l.=oryzias latipes, b.t.=bos taurus. *In situ* hybridisation expression of (B–D′) *pthlha*, (E–E″*)gdf5,* (F–H′) *Col9a2* and (I–J′) *Col10a1* B, G, H, I and J show lateral views; B′, C′,G′,H, I″ and J′ ventral views. D–F″ are cryosections. All images are orientated with anterior to left. B,B′, G, G′, I and I′ are 72 hpf, E is 96 hpf, C–D′, E′–F′, H, H′, J and J′ are 120 hpf. Ba = branchial arches, cl = cleithrum, m = maxilla, ch = ceratohyal, op = operculum, ps = parasphenoid, pn = pronephros, 5ba = 5th branchial arch, mc = Meckel's cartilage, pf=pectoral fin.

Expression of PTHrP/PTHLH has been shown to be increased at the protein level in cartilage from patients with OA^28^, although the level of the Pthrp at the transcript level has been shown to be decreased in surgically induced OA in the rat^29^. We identified two possible zebrafish homologues of PTHLH; to establish which is the closest homologue we generated a phylogenetic tree [Fig. 2(A)] and established that the homologue which we denote as *pthlha* is more closely related to other vertebrate *Pthlh* genes than the gene which we denote *pthlhb*. In zebrafish *pthlha* was expressed at 72 hpf in the pectoral fins and in the cartilages that form the ventral jaw [Fig. 2(B)]. At 120 hpf, *pthlha* expression could be seen in the operculum [Fig. 2(C, C′)], and by this stage jaw cartilage expression of *pthlh* was mainly restricted to the 5th branchial arch (a cartilaginous structure which is subsequently mineralised) at the position where the teeth will later form. Additionally, strong expression was seen in the mesonephros pronephros [Fig. 2(C)].

### *Gdf5* expression

GDF5 variants have been demonstrated to show association with human OA^10,30^. In mouse, Gdf5 becomes restricted to joints and is required for correct joint specification^31^. In zebrafish, *gdf5* expression has been previously described in pharyngeal arch cartilages up to 96 hpf^20^. *Gdf5* is also expressed in the developing fin joints through larval and early adult life^32^. At 96 and 120 hpf we saw expression restricted to the jaw joints that form between the Meckel's cartilage and palatoquadrate and the tip of the ceratohyal [Fig. 2(E–E″)], suggesting that it has a conserved function in the specification of joints in zebrafish.

### Collagen expression

We here describe the expression of three collagen types involved in skeletal development (*col2a1, col9a1,* and *col10a1*), and we have generated and analysed two transgenic reporter lines (*col10a1* and *col2a1*) *in vivo*. These specific collagens were chosen on the basis that all are expressed in chondrocytes at specific stages of maturation during endochondral ossification^12^.

Mutations in COL9A2 have been linked by numerous groups to autosomally dominant multiple epiphyseal dysplasia (MED), a disease which manifests from childhood with axial limb deformities, joint pain and gait abnormalities and predisposes sufferers to early onset OA^33^. In zebrafish, *col9a2* was expressed strongly in the otic capsule of the ear at 72 hpf, and was more weakly detected in the jaw cartilage elements [Fig. 2(G–G′)]. At 120 hpf, *col9a2* transcripts were present throughout cartilagenous structures of the ventral jaw and remained strongly expressed in the otic capsule [Fig. 2(F–F’, H–H′)].

Col10a1 expression is a hallmark of chondrocyte hypertrophy in mammals^34^. In zebrafish, at 72 hpf, *col10a1* was expressed in the cleithrum, operculum, and parasphenoid, bone elements that form through intramembranous ossification [Fig. 2(I–I′)]. At 120 hpf, *col10a1* was still strongly expressed in dermal bone elements, but could, at this stage of development, also be detected in chondral bone elements such as the centre of the ceratohyal [Fig. 2(J–J′)].

### The col10a1 transgenic reporter line allows visualisation of chondrocyte hypertrophy in living fish

**Fig. 3:**
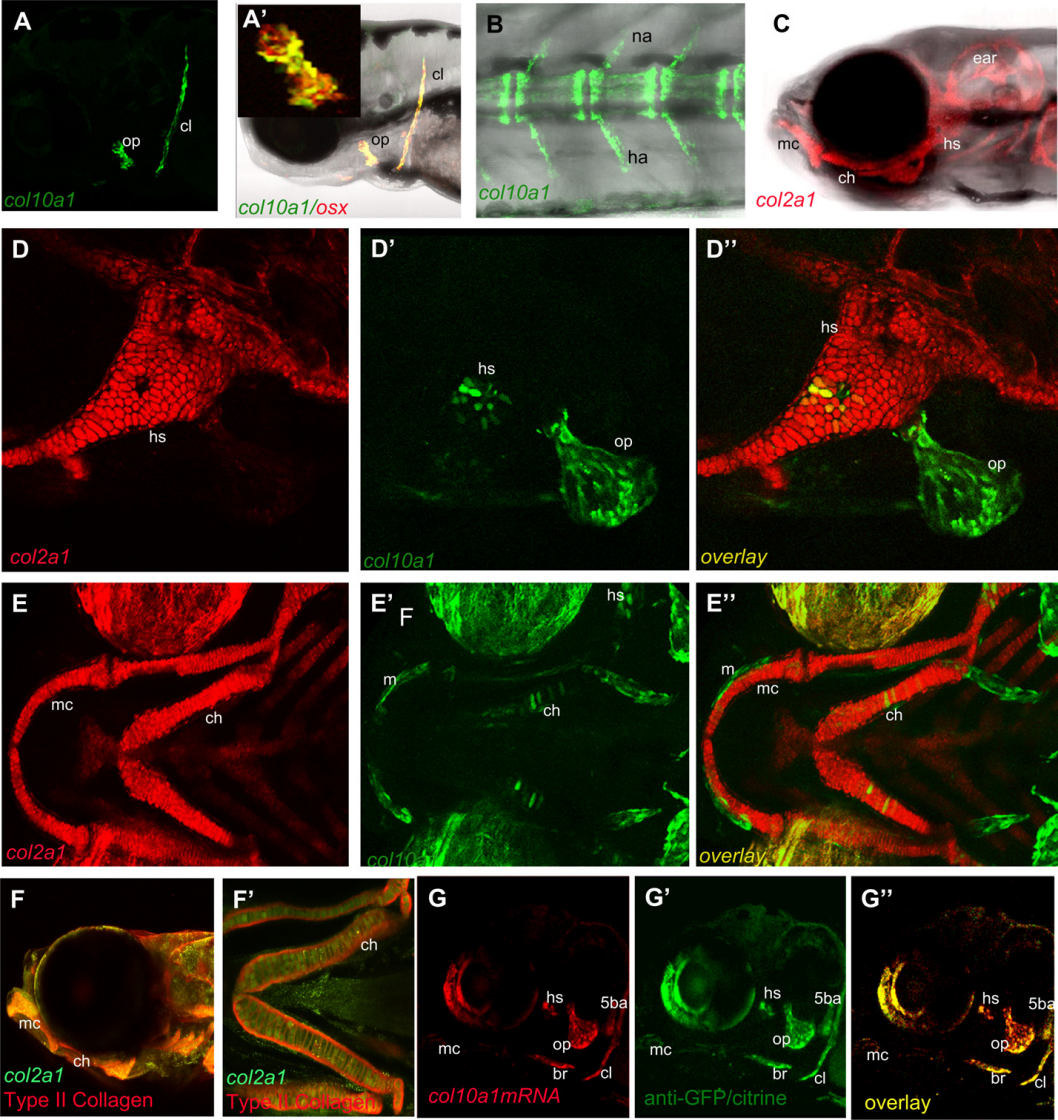
***Col10a1, osterix* and *col2a1* transgenic reporter expression** expression of tg(*col10a1*BAC:citrine) A, A′ B, C, E′, E″, F′ and F″, tg(*col2a1*BAC:mcherry) C, D, D″, E′ and E″ and Tg(OlSp7:mCherry)zf131 A′(A) Col10a1BAC:citrine reporter expression in live zebrafish at 72 hpf, (A,A′), 120 hpf (D–E″) and 14 dpf (B). B is of the vertebral column at the level of the cloaca. (F and F′) 96 hpf col2a1BAC:mCherry transgenic larvae fixed and labelled with anti-mCherry to detect transgene activity in green and with II–II3B3 antibody (DSHB) which detects type II collagen protein in red, green labelled cells with transgene activity are surrounded by matrix positive for collagen 2 protein. G) Fluorescent *in situ* for *col10a1* mRNA detected with anti-digoxigenin rhodamine. G′) anti-GFP/citrine staining and G″ shows the overlay of G and G′, showing that all regions that stain for *col10a1* mRNA are also positive for the fluorescent transgenic protein. A, A′, D–D″ F and G–G″ are lateral views and E–E″ and F′ are ventral. All are projections of confocal stacks, all presented with anterior facing left. cl = cleithrum, m = maxilla, ch = ceratohyal, op = operculum, mc = Meckel's cartilage, 5ba = 5th branchial arch and teeth, hs = hyosymplectic, br = branchiosteal rays, na = neural arches, ha = haemal arches.

The *col10a1BAC*:citrine transgene recapitulated the expression of the mRNA expression seen by *in situ* hybridisation [Compare Fig. 3(A) with Fig. 2(I–I′)] and can be used to track expression of the gene in live fish by fluorescence microscopy (Fig. 3 A, B, D′–D″ and E′–E″). At 72 hpf expression could be seen in the operculum and cleithrum [Fig. 3(A–A′)] and colocalises with expression of an osteoblast reporter Tg(Ola.Sp7:NLS-GFP)zf132 [Fig. 3(A′) [inset shows the operculum^24^], thus confirming as previously reported that *col10a1* is expressed in osteoblasts as well as in chondrocytes during zebrafish^21^, and gar^35^ development. At later stages of development, (shown here at 14 dpf), *col10a1* was expressed throughout the vertebral column [Fig. 3(B)], including the neural and hemal arches [Fig. 3(B)]. C*ol2a1*:*mCherry* was expressed in all chondrocytes during early skeletal development [Fig. 3(C–E)], while at later stages *col10a1* becomes expressed in a subset of chondrocytes as they maturate to hypertrophy [Fig. 3(D–E) and data not shown]. As shown in Fig. 3(D″ and E″), in a double transgenic line Tg(col10a1BAC:mcitrine)^hu7050^; Tg(*col2a1aBAC*:mCherry)^hu5900^ it was easy to distinguish the less mature non-hypertrophic chondrocytes from the chondrocytes entering hypertrophy (which appear yellow in overlays and are located within the cartilage element).

### Transgenic reporter fish faithfully recapitulate the endogenous expression of col2a1a and col10a1

We generated a stable transgenic line marking chondrocytes by injection of the col2a1a:BAC mCherry construct previously described^18^. In this line (also described in^23^ mCherry labels all chondrocytes at early stages of development. To demonstrate that mCherry expression marks cells which express col2a1a we used immunohistochemistry to detect the mCherry protein and to detect the Collagen II protein. As expected the cells which express col2a1a:mCherry are surrounded by matrix containing the Collagen II protein [Fig. 3(F, F′)]. To demonstrate that the col10a1 transgenic line recapitulates the expression of endogenous *col10a1* mRNA we performed an *in situ* for *col10a1*, in 5 dpf larvae, which we detected with an anti-digoxigenin-rhodamine secondary antibody and immune labelled for GFP/citrine which we detected with 488-conjugated secondary antibody [Fig. 3(G)]. The overlay of the two antibodies demonstrates that all regions in which col10a1 mRNA is present show co-expression of citrine [Fig. 3(G–G″)].

## Discussion

### Osteoarthritis associated genes are dynamically expressed during zebrafish development

In this study, we describe the spatiotemporal expression of the OA associated gene *mcf2l* during early development. Mcf2l (previously identified as Ost and Dbs) is a guanine nucleotide exchange factor, which in purified form, catalyses nucleotide exchange on RhoA and Cdc42^36^. In 5-week-old rat brain sections, Ost/Mcf2l expression was seen in neurons and α-tanycytes^36^. However, to date, the developmental expression of Mcf2l is unknown. Functionally, Mcf2l has been shown to stimulate migration of breast carcinoma cells and of Schwann cells^37,38^. Furthermore, Mcf2l has been identified, through genome-wide association (GWA) studies, to have a significant association with OA. However, with only limited expression and functional data, it is currently unclear how the genetic variants contribute to OA, although the fact that it plays a role in Schwann cell migration has lead to predictions that it could be involved in the pain response to OA^38,39^.

We demonstrate that *mcf2l* is dynamically expressed in a range of cell types during development, including Kupffer's vesicle. We also observe diffuse expression of *mcf2l* in the brain throughout development, consistent with the strong expression seen in the brain in rat^36^. Importantly, we have also observed expression of *mcf*2l in the developing jaw cartilages, which suggests that mcf2l has a function in cartilage development, offering another potential explanation for how MCF2L could play a role in OA.

Additionally, we have characterised the expression pattern of *pthrp* in zebrafish. PTHrP has been shown to regulate the entry of chondrocytes into hypertrophy, and application of PTHrP can block the ability of RUNX2 to induce expression of hypertrophic markers such as COL10A1 in culture^40^. Here, we show that *pthrp* is dynamically expressed during zebrafish development. Pthrp is only transiently expressed in cartilage elements, becoming restricted to the 5th branchial arch, adjacent to where the first tooth will attach to the arch at 120 hpf^41^. PthrP is required for tooth eruption in mice^42^, suggesting a likely conservation of function between teleosts and tetrapods. We also observed strong expression of *pthrp* in the developing mesonephros. No significant overlap in expression is seen with the zebrafish parathyroid hormones *pth1* and *pth2,* whose expression is limited to the lateral line and sense organs^43^. *Gdf5*, in zebrafish, becomes restricted to the site of joints in the developing cartilage as is the case in mice^31^. Additionally, a number of other genes implicated in human OA such as FRZB/sfrp3^44^ and ASPN^45^ show expression patterns that hint at conserved functions in zebrafish^46^ (data available on zfin.org).

### Collagens

Various collagens have been implicated in OA susceptibility and pathogenesis; mutations in *col1a1* are associated with osteogenesis imperfecta both in the zebrafish and in humans^47,48,49^. Only weak association has been seen between COL1A1 and OA through GWAS^56^, but other studies report increased levels of COL1A1 in osteoblasts from OA^51^, suggesting a shift towards an osteophytic phenotype. Type II Collagen breakdown fragments are frequently used as a biomarker for OA, while synthesis of the pro collagen gene COL2A1 has been shown to be increased in many models of OA (see for example review by Garvican et al^52^). COL9A2 has been linked through candidate gene association studies to OA of the hip^53^. Ectopic chondrocyte hypertrophy is seen as a hallmark of OA^14^, and Col10a1 expression is the best characterised marker of hypertrophic chondrocytes^54^. We show here that all four collagen genes show dynamic expression in the developing skeleton of the zebrafish and that in zebrafish, as in mammals, col10a1 can be used as a marker of hypertrophic chondrocytes in addition to marking zebrafish osteoblasts.

### Zebrafish as a model for OA

There are many existing animal models established for the study of OA ranging from small rodents to large mammals such as sheep^1^. One might think, therefore, that there is little point in adding another model to the list. Zebrafish, although their skeleton is subject to different loading due to their aqueous environment, do have a number of key advantages for studies of OA genetics that may complement those in existing animal models. One example is that, due to their translucency during larval development, organogenesis, even deep tissues such as the skeleton can be viewed microscopically *in vivo*. In the case of transgenic reporter lines, such as the ones described in this manuscript, detailed observations on the location and behaviour of the cells expressing the genes in live fish can be made, something which is not possible in the existing animal models, for which the depth and mineralisation of the joints limits the options for imaging. Already a number of zebrafish cartilage skeletal mutants have been demonstrated to share pathology with human disease; these include the heparin sulphate proteoglycan mutants such as *ext2/dak* and *papst/pic* which model the human osteosarcoma condition Multiple Hereditary Exostoses (HME)^50,55,56,57^. The ongoing generation of an increasing number of transgenic lines marking bone via promoters such as *osterix*^24,58,59^ and *osteocalcin*^60^ or cartilage by *col2a1*^28^ and *col10a1* (described here) as well as reporter lines demonstrate activity of major signalling pathways (reviewed in Hammond and Moro^23^) will further benefit fish skeletal research.

It has previously been shown that all five of the genes in the OA susceptibility locus on 7q22 are expressed in zebrafish, and two of these, *cog5* and *dus4l* show expression in cartilage at 5 dpf^9^. Therefore, taken together with the published expression of *frzb/sfrp3*^61^, asporin (direct data submission to zfin.org), and the expression patterns detailed in this manuscript, candidate OA genes identified through GWAS have homologues in zebrafish, and most have been shown to be expressed in the skeletal tissues at various stages of development. The expression of these ‘OA’ genes in zebrafish can be seen in the developing skeletal system and it is likely that they will have conserved functions during skeletogenesis with their mammalian homologues. An advantage of studying the function of OA genes in zebrafish is the relative ease of genetic manipulation in the zebrafish, such as morpholinos for transient knockdown of protein translation^67^ and transgenic approaches for overexpression of genes of interest, either globally or under the control of a promoter of interest. There have also been recent advances in the ability to rapidly generate stable mutant lines^63,64,65^ with zinc finger nucleases (ZFNs) or transcription activator-like effector nucleases (TALENs); together these techniques allow the functions of genes to be dissected *in vivo*. This is essential, as functional analyses demonstrating that zebrafish can develop pathologies relevant to OA will be required to establish zebrafish as a model for OA.

A major stumbling block in the management of OA is the paucity of pharmaceutical therapies. The development of zebrafish tools relevant to the study of OA raises the prospect of using zebrafish for compound screens; the protocols for such screens are well described in zebrafish^62,66^. These have the potential to identify novel modifiers of cartilage and bone phenotypes; streamlining the path into drug discovery programmes to test for compounds with therapeutic properties in OA and related diseases. As expression of type X Collagen is one of the hallmarks of chondrocyte hypertrophy, double transgenic lines of zebrafish carrying reporters for both type II Collagen tg(*col2a1*BAC:mCherry)^hu5900^ and Type X Collagen tg(*col10a1*BAC:citrine)^hu7050^, as described here, could be used in drug screens to identify compounds that can block the onset of chondrocyte hypertrophy and thus potentially to prevent OA progression.

In summary, we have described the developmental expression of a number of genes with relevance to OA in the zebrafish and we describe a new transgenic reporter line for Type X collagen, which can be used to study chondrocyte hypertrophy in live fish. This can be used as a platform for further research into the functions of the OA genes described here in the developing skeleton and may have uses in large scale screening programmes.

## Author contributions

CLH and SSM designed and conceived the study, CLH conducted experiments and wrote the manuscript. REM, CS, LHB, REHS and LFAH performed and analysed experiments.

The research was funded by an Arthritis Research UK career development award to CLH. SSM is supported by FP7 TREAT_OA, and also gratefully recognizes the support of the Smart Mix Programme of the Netherlands Ministry of Economic Affairs and the Netherlands Ministry of Education, Culture and Science.

## Conflicts of interest

The authors report no conflicts of interest.

## Figures and Tables

**Table I:** Primers used for cDNA amplification

Gene name	Forward primer sequence	Reverse primer sequence	Ensembl gene ID	Transcription
*pthrpa/pthlha*	CGAACGCTGCAGGATTTTA	AAGGTCAGCAGCACCTTGAT	**ENSDARG00000031737**	T7
*mcf2la*	GAGAAAGCCCCGTCATACAG	AATTAACCCTCACTAAAGGGAGTTTCTTCCCTCCCTCATCCT	**ENSDARG00000075859**	T3 (t3 pol site in reverse primer)
*col9a2*	AGGTGCTACCGGAATGATTG	GGATCCATTAACCCTCACTAACGGGAGGTCCAGGTCGTCCTG	ENSDARG00000024492	T3 (t3 pol site in reverse primer)
